# Healthcare costs in patients with metastatic lung cancer receiving chemotherapy

**DOI:** 10.1186/1472-6963-11-305

**Published:** 2011-11-10

**Authors:** Montserrat Vera-Llonch, Derek Weycker, Andrew Glass, Sue Gao, Rohit Borker, Beth Barber, Gerry Oster

**Affiliations:** 1 Policy Analysis Inc (PAI), Four Davis Court, Brookline, MA, USA 02445; 2 Kaiser Permanente Northwest, 3800 N. Interstate, Portland, OR, USA 97227; 3 Global Health Economics, Amgen Inc., One Amgen Ctr. Drive MS28-3D, Thousand Oaks, CA, USA 91320

## Abstract

**Background:**

To characterize healthcare resource utilization and costs in patients with metastatic lung cancer receiving chemotherapy in the US.

**Methods:**

Using data from a large private multi-payer health insurance claims database (2000-2006), we identified all patients beginning chemotherapy for metastatic lung cancer. Healthcare resource use (inpatient, outpatient, medications) and costs were tallied over time from date of therapy initiation ("index date") to date of disenrollment from the health plan (in most instances, presumably due to death) or the end of the study period, whichever occurred first. Healthcare utilization and costs were characterized using Kaplan-Meier sample average methods.

**Results:**

The study population consisted of 4068 patients; mean (SD) age was 65 (11) years. Over a median follow-up of 334 days, study subjects averaged 1.5 hospital admissions, 8.9 total inpatient days, and 69 physician office and hospital outpatient visits. Mean (95% CI) cumulative total healthcare costs were $125,849 ($120,228, $131,231). Costs of outpatient medical services and inpatient care constituted 34% and 20% of total healthcare costs, respectively; corresponding estimates for outpatient chemotherapy and other medication were 22% and 24%.

**Conclusion:**

Our study sheds additional light on the burden of metastatic lung cancer among patients receiving chemotherapy, in terms of total cost thru end of life as well as component costs by setting and type of service, and may be useful in informing medical resource allocation in this patient population.

## Background

Lung cancer is a common and aggressive disease that is usually diagnosed in relatively late stages with little or no chance of cure. The American Cancer Society estimated that there would be about 219,440 new cases of lung cancer in the US in 2009, accounting for approximately 15% of all new cancer diagnoses [1]. More than 45% of all patients with incident lung cancer present with advanced disease [2]; median survival time among these patients ranges from 8 to 13 months [3]. There are about 160,000 deaths annually due to lung cancer in the US, surpassing the total number of deaths from breast, prostate, and colorectal cancer combined. In 2004, costs of care in patients with lung cancer were estimated to account for approximately 20% ($4.2 billion) of all Medicare expenditures for the treatment of cancer, a figure that is greater than the estimated total cost of treatment among patients with colorectal or prostate cancer ($2 billion) [4].

Metastatic lung cancer is difficult to treat. Systemic chemotherapy--often in combination with targeted therapies, such as bevacizumab--is currently the preferred treatment strategy [5] for lung cancer patients with non-squamous histology. However, such treatment typically produces only modest improvements in survival and symptom palliation. Median survival among patients receiving bevacizumab--the most efficacious treatment at this time--is about 12 months.

Because the benefits of chemotherapy for metastatic lung cancer -- in terms of both extensions in life expectancy and enhanced quality of life -- are typically limited, the cost of such treatment (as well as associated follow-on care) is an especially important consideration in an era of increased emphasis on achieving an acceptable balance between the costs and benefits of medical interventions [6]. While a few retrospective longitudinal studies [7-11] have estimated the cost of metastatic lung cancer in the US, these studies employed varied designs and methods (i.e., in terms of patient populations, disease definitions, and measure of healthcare costs), did not track lifetime healthcare resource use and costs, or did not analyze cost components by setting or type of service. Up-to-date data on resource use and costs among patients with metastatic lung cancer -- overall and by constituent component -- thus may help inform current decision-making about the optimal allocation of healthcare resources.

Contemporary data on resource use and costs in this patient population also may help inform cost-effectiveness evaluations of new strategies for the prevention, screening, and treatment of early stage and metastatic lung cancer; such information increasingly plays a role in regulatory and reimbursement decision making [6]. Evaluations of early stage interventions, for example, typically consider the economic consequences of disease progression (i.e., treatment failure), which may be characterized using data on levels of resource use and costs among patients with metastatic lung cancer. We therefore used a large US private health insurance claims database to estimate cumulative healthcare resource utilization and costs through end of life in patients receiving chemotherapy for metastatic lung cancer.

## Methods

### Data Source

Data for this study were obtained from the MarketScan Commercial Claims and Encounters Database, a large private health insurance claims database, and spanned the period January 1, 2000 through December 31, 2006. The database is comprised of medical (i.e., facility and professional service) and outpatient pharmacy claims from employer-sponsored health insurance plans covering more than 10 million persons annually, including employees as well as their spouses and dependents. The plans provide health benefits under a number of different products, including fee-for-service and capitated (full, partial) systems. Plan members reside throughout the US; approximately 10% are aged 65 years or older.

Data available for each facility and professional-service claim include date and place of service, diagnoses (in International Classification of Diseases, Ninth Edition, Clinical Modification [ICD-9-CM] format), procedures performed/services rendered (in Health Care Financing Administration Common Procedure Coding System Level II [HCPCS], Common Procedural Terminology [CPT], ICD-9-CM, and Uniform Bill-92 [UB-92] formats), and quantity of services (professional-service claims only). Data available for each retail pharmacy claim include the drug dispensed (in National Drug Code [NDC] format), dispensing date, quantity dispensed, and number of days of therapy supplied. All claims include paid (i.e., reimbursed) amounts, including patient deductibles, copays, and/or coinsurance amounts. Selected demographic and eligibility information is also available for persons in the database, including age, sex, geographic location, coverage type, and the start and end dates of health insurance coverage. Patient-level data can be arrayed chronologically to provide a detailed longitudinal profile of all medical and pharmacy services received.

All patient-identifying information has been either fully encrypted or removed from the database; it is therefore compliant with the Health Insurance Portability and Accountability Act of 1996 and federal guidance on Public Welfare and the Protection of Human Subjects. Per the Code of Federal Regulations (45 CFR 46 §46.101), IRB review was not needed for a study of this nature, since ". . . subjects cannot be identified, directly or through identifiers linked to the subjects . . .".

### Study Subjects

The study population consisted of all patients aged ≥ 18 years who initiated chemotherapy for metastatic lung cancer between January 1, 2000 and December 31, 2006, and who met all other inclusion criteria. Subjects were selected for inclusion in the study population as follows.

First, we identified all patients who had two or more healthcare encounters (on different days) with a diagnosis of lung cancer (ICD-9-CM 162.x, V10.1x) during the period of interest; all such patients were designated as having "lung cancer". Second, from among these patients, we identified all those who also had two or more encounters with a diagnosis of distant secondary malignant neoplasm (ICD-9-CM 196.2, 196.5, 196.8, 197.1-199.0) during this period; this subgroup of patients was designated as having "metastatic lung cancer". (We required two or more encounters for both lung cancer and metastatic disease to increase the specificity of our algorithm [i.e., reduce the number of false-positives].) Third, from among the subgroup of patients with metastatic lung cancer, we identified all those with any evidence of receipt of chemotherapy based on a procedure code for chemotherapy administration or receipt of a chemotherapy agent (codes available upon request), beginning 45 days prior to the date of the earliest encounter with a diagnosis of secondary malignant neoplasm. (A 45-day window was employed to capture instances where chemotherapy might have been initiated prior to the first notation of metastatic disease on a health insurance claim.) The date of initial receipt of chemotherapy was designated the "index date".

To minimize the possibility of including patients who may have received chemotherapy for primary tumors other than lung cancer, we excluded all patients with two or more encounters with a diagnosis of a primary malignant neoplasm other than lung cancer (ICD-9-CM 140-161, 163-172, 174-195, 200-208) 61 or more days prior to their first evidence of metastatic disease, unless the site of the other primary neoplasm and the site of metastases were the same (e.g., malignant neoplasm of bone [170.0] and metastasis to bone [198.5]). (The last exclusion was used to account for instances where a site of metastatic involvement might have been miscoded as a primary tumor.) The only exception was patients with malignant neoplasm of the skin, whom we retained in the sample because the skin is not a site of metastatic involvement in lung cancer. To ensure completeness in case ascertainment, we also excluded patients if they were not continuously eligible for comprehensive health benefits during the 12-month period preceding their index date.

### Follow-Up

Follow-up began on the index date and ended with disenrollment from the health plan (in most instances, presumably due to death) or the end of the study period, whichever occurred first.

### Measures

Healthcare utilization was assessed in terms of the percentage of patients receiving inpatient services, outpatient services, and outpatient medications (i.e., drugs administered in an outpatient setting or dispensed at an outpatient pharmacy), and corresponding levels of care provided. Healthcare costs were estimated using paid amounts, and were characterized on an overall basis and by component of care (i.e., inpatient and outpatient services, outpatient medications). Utilization and costs of outpatient services were further stratified by setting of care (i.e., emergency room, physician office, hospital outpatient, home health/hospice/skilled nursing facility [SNF], and other), and by type of service (e.g., evaluation and management, laboratory, radiology diagnostic, etc.) within selected settings, as feasible. Utilization and costs of medications were tallied on an overall basis as well as for distinct medication groups. Cost of chemotherapy administration was tallied in the outpatient services category.

### Analyses

Characteristics of study subjects were examined, including age, geographic region, payer, prevalence of selected pre-existing comorbidities, and healthcare expenditures up to 12 months preceding the index date. Age, geographic region of residence, and payer type were ascertained as of the index date. Comorbidities were ascertained based on the presence of relevant diagnosis codes during the history period.

Cumulative total healthcare utilization and costs were tallied for each patient on a daily basis from the index date through the end of follow up. Mean cumulative utilization and costs were calculated using Kaplan-Meier Sample Average (KMSA) methods. Using this technique, the follow-up period for each patient was partitioned into one-month intervals and Kaplan-Meier estimates of the probability of survival and continued health plan enrollment to the beginning of each interval were calculated. Expected utilization and associated costs of care were then calculated as the sum of the Kaplan-Meier estimates of the probability of survival to the beginning of each interval multiplied by corresponding estimates of utilization and costs respectively during the interval conditional on survival to the beginning of the interval [12]. Survival probabilities were calculated using dates of disenrollment, as this was assumed to occur primarily as a result of death in this patient population; subjects who were observed through the end of the study period (i.e., December 31, 2006) were censored as of this date. Ninety-five percent confidence intervals (95% CIs) were calculated for total costs using techniques of nonparametric bootstrapping [13]. Significance testing was not performed, as there were no *a priori *hypotheses. Cumulative total healthcare costs also were estimated focusing on patients who were not censored, and thus for whom claims data were available from their date of diagnosis through date of death (i.e., plan disenrollment); component costs were described among these patients as well.

## Results

### Patient Characteristics

The study population consisted of 4068 patients; subjects excluded due to failure to meet study entry criteria are provided in the Additional File 1. Mean (SD) age was 65 (11) years; about 84% of patients were older than 55 years (Table 1). Mean (SD) healthcare costs up to 12 months preceding the index date were estimated $28,562 ($31,136). Mean (SD) duration of follow-up was 500 (488) days (median = 334 days or ~ 11 months).

**Table 1:** Demographic and clinical characteristics of study subjects with metastatic lung cancer receiving chemotherapy

Parameter	Value	
	**n = 4068**	
Age (n,%)		
18-34	20	0.5
35-44	108	2.7
45-54	513	12.6
55-64	1,481	36.4
> = 65	1,946	47.8
Geographic region (n,%)		
Northeast	538	13.2
Northcentral	1,249	30.7
South	2,071	50.9
West	208	5.1
Payer type (n,%)		
HMO	81	2.0
Indemnity	1,814	44.6
PPO	1,210	29.7
POS	941	23.1
Other	21	0.5
Comorbidities (n,%)		
Cerebrovascular Disease	426	10.5
Coronay heart disease	910	22.4
Heart failure	66	1.6
Peripheral arterial disease	165	4.1
Diabetes	808	19.9
Kidney Disease	60	1.5
Liver Disease	272	6.7
Respiratory Disease	2,384	58.6
Health care expenditures prior to index date (mean, SD)		
-365 days	28,562	31,136

### Healthcare Utilization

Seventy-two percent of patients were admitted to an acute-care hospital at least once; the mean number of hospital admissions was 1.5, and the mean number of total inpatient days was 8.9 (Table 2). Patients also averaged 41.5 physician office visits, 1.6 emergency room visits, 18.2 hospital outpatient visits, 4.7 home health/hospice/SNF visits, and 10.1 other outpatient encounters. In addition to evaluation and management services, use of outpatient services was highest for radiology diagnostic services (91%), followed by laboratory (82%), mental health services (68%), anesthesia and invasive procedures (63%), radiation therapy (55%), and supplies (52%). Patients averaged 39.7 medication prescriptions during follow-up. Use of outpatient medication was highest for the combined category, analgesics, sedatives, and antidepressants (89%), followed by anti-emetics (85%), anti-infectives (74%), gastrointestinal drugs (other than anti-emetics) (57%), and cardiovascular drugs (53%). Fewer than one-half of study subjects received fluids and electrolytes (49%), erythropoietin stimulating agents (ESAs) (46%), blood products and anticoagulants (34%), granulocyte colony stimulating factors (29%), and bisphosphonates (18%), respectively.

**Table 2:** Utilization of healthcare among patients with metastatic lung cancer receiving chemotherapy

	Value	
	n = 4068	
Inpatient, mean		
Acute hospital		
Admissions (#)	1.5	
Days (#)	8.9	
Outpatient Services, mean		
Physician Office (#)	41.5	
Emergency Room (#)	1.6	
Hospital Outpatient (#)	18.2	
Home Health (#)	4.7	
Other (#)	10.1	
Pharmacy Prescriptions (#), mean	39.7	
Inpatient, n (%)		
Acute hospital	2,933	(72.1%)
Outpatient Services*, n (%)		
Emergency Room	2,532	(62.2%)
Physician Office/Hospital Outpatient		
Evaluation and Management	3,947	(97.0%)
Laboratory	3,331	(81.9%)
Radiology Diagnostic	3,691	(90.7%)
Radiology Therapeutic	2,241	(55.1%)
Nuclear Medicine	1,826	(44.9%)
Anesthesia and invasive procedures	2,581	(63.4%)
Blood & Transfusion	338	(8.3%)
Physical & Occupational Therapy	402	(9.9%)
Medical & Surgical Supplies	2,104	(51.7%)
Mental health-care	2,772	(68.1%)
Other	134	(3.3%)
Chemo administration	3,943	(96.9%)
Subtotal	4,032	(99.1%)
Home Health/Hospice/Skilled Nursing	2,200	(54.1%)
Other	3,314	(81.5%)
Total	4,053	(99.6%)
Medication, n (%)		
Chemotherapy	4,068	(100.0%)
G-CSF	1,159	(28.5%)
ESAs	1,859	(45.7%)
Pain, sedatives, antidepressants	3,629	(89.2%)
Anti-infectives	2,991	(73.5%)
Anti-emetics	3,445	(84.7%)
Bisphosphonates	721	(17.7%)
Biologics	186	(4.6%)
Gastrointestinal	2,337	(57.4%)
Electrolytes, Caloric, Water	1,981	(48.7%)
Cardiovascular	2,161	(53.1%)
Blood products & Anticoagulants	1,393	(34.2%)
Other	3,976	(97.7%)
Total	4,068	(100.0%)
TOTAL	4,068	(100.0%)

*Other than medication administration

### Healthcare Costs

**Figure 1:**
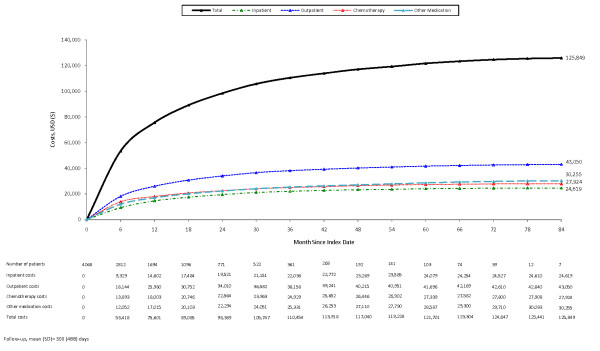
**Cumulative costs among patients with metastatic lung cancer receiving chemotherapy**.

**Figure 2:**
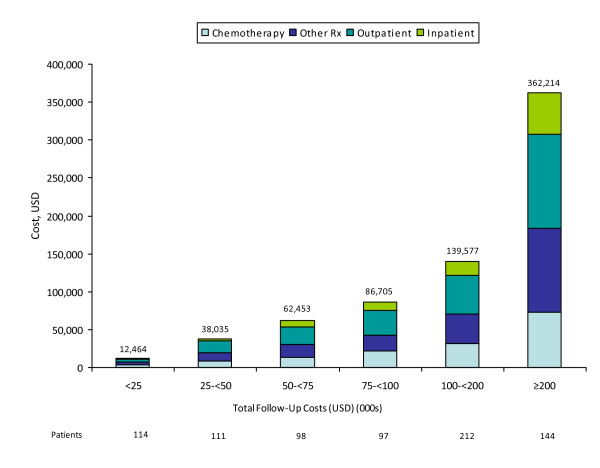
**Component costs of care among patients with metastatic lung cancer receiving chemotherapy, by total cost of care during follow-up**.

Mean (95% CI) cumulative healthcare costs averaged $125,849 ($120,228, $131,231) per patient over a mean follow-up period of 500 days (Figure 1). Outpatient care and inpatient care constituted 34% and 20% of total healthcare costs, respectively; corresponding estimates for outpatient chemotherapy and other medication were 22% and 24% (Table 3). Cumulative healthcare costs ($131,344) among non-censored patients (n = 776) were consistent with those reported for the full sample. About one-half (54%) of non-censored patients had total costs less than $100,000 (Figure 2); outpatient and inpatient services accounted for approximately one-half (49%) of total healthcare costs, while chemotherapy accounted for about 24% of the total. Among the remaining non-censored patients with total costs exceeding $100,000, corresponding figures were 50% and 22%.

**Table 3:** Cumulative cost of medical-care services among patients with lung cancer receiving chemotherapy

	Mean (USD)(%)	
	n = 4068	
Inpatient		
Acute hospital	24,619	19.6%
Outpatient Services*		
Emergency Room	802	0.6%
Physician Office/Hospital Outpatient		
Evaluation and Management	2,436	1.9%
Laboratory	1,227	1.0%
Radiology Diagnostic	6,881	5.5%
Radiology Therapeutic	6,054	4.8%
Nuclear Medicine	838	0.7%
All Surgery/Anesthesia	1,654	1.3%
Blood & Transfusion	144	0.1%
Physical & Occupational Therapy	143	0.1%
Medical & Surgical Supplies	697	0.6%
Chemo administration	2,054	1.6%
Mental health-care	25	0.0%
Other	14,274	11.3%
Subtotal	36,426	28.9%
Home Health/Hospice/Skilled Nursing	1,531	1.2%
Other	4,291	3.4%
Total	43,050	34.2%
Medication		
Chemotherapy	27,924	22.2%
G-CSF	2,968	2.4%
ESAs	4,526	3.6%
Pain, sedatives, antidepressants	1,693	1.3%
Anti-infectives	345	0.3%
Anti-emetics	2,500	2.0%
Bisphosphonates	1,133	0.9%
Biologics	2,783	2.2%
Gastrointestinal	536	0.4%
Electrolytes, Caloric, Water	58	0.0%
Cardiovascular	606	0.5%
Blood products & Anticoagulants	976	0.8%
Other	12,132	9.6%
Total	58,179	46.2%
TOTAL	125,849	100.0%

*Other than medication administration

## Discussion

Using a large private health insurance claims database, we examined healthcare utilization and costs among patients with metastatic lung cancer receiving chemotherapy. Over a median follow-up of 334 days, healthcare costs averaged $125,849 per patient. Chemotherapy and other outpatient medication accounted for 22% and 24% of total costs, respectively; other outpatient and inpatient services accounted for 34% and 20% of these costs, respectively.

Comparisons of our findings with prior published estimates are not straightforward due to differences in the characteristics of patients included in these studies as well as the methodologies employed. Also, more recent increases in spending for advanced lung cancer may reflect not only changes in detection and staging techniques and management over time, but also increased use of chemotherapy and targeted therapies. Three studies were identified that examined the cost of advanced lung cancer using the linked SEER-Medicare claims data [4,7,9]. Yabroff and colleagues reported that costs of care during the last year of life among patients with distant lung cancer averaged $85,392 (in 2010 USD); hospitalization costs were the single largest component of cost among these patients. This estimate, however, represented cost of patients dying from lung cancer as well as those with lung cancer who died of other causes, and did not include the cost of outpatient prescription medications. Similar findings were reported by Lang et al., who used linked SEER-Medicare claims data to estimate costs among patients receiving first-line doublet chemotherapy. Costs (excluding those of chemotherapy) were reported to average $85,174 (in 2010 USD); hospitalization and physician visits represented more than 85% of the total. The third study [9] reported lifetime costs among patients with distant disease to be $49,971 (in 2010 USD), but it should be noted that the study was conducted in the mid-1990s. Other studies have included one conducted at Henry Ford Health System, which reported an estimate of $44,770 (in 2010 USD) [8] for resources consumed between first progression and death or end of study among patients with Stage IIIB or IV disease who received chemotherapy; about one-half of that cost was associated with inpatient services, followed by outpatient, emergency and pharmacy services. Finally, two additional studies reported estimates of $40,226 (in 2010 USD) (excluding chemotherapy costs) and $12,584 (in 2010 USD), respectively, for the last six months of life among patients in a Veteran Affairs medical center [10] and in the terminal phase of their disease [11], respectively. The majority of published studies did not track the full complement of lifetime costs among patients with metastatic lung cancer. We believe this is one of the primary reasons why our estimate of mean healthcare costs ($125,849) is substantially higher than those previously published. Prior studies also did not report on the individual components of healthcare utilization and costs, which was an important objective of our study.

Caution should be exercised in generalizing from the results of our study to other patient populations and settings. First, our study employed data from a large US private health insurance database comprising information from employer-sponsored health insurance plans on resource utilization and costs of active employees, early retirees, and their dependents along with Medicare-eligible retirees with employer-sponsored supplemental Medicare coverage. Because such persons may differ systematically from other patients with metastatic lung cancer (e.g., the elderly with traditional Medicare fee-for-service coverage, and the uninsured, who are not represented in our database)--in terms of health status and/or levels of resource utilization and costs--findings of similar analyses may differ in other patient populations. Second, our study used a novel algorithm for patient selection, the accuracy of which is unknown. Cooper and colleagues reported that the sensitivity and positive predictive value of using ICD-9-CM diagnosis codes with healthcare claims to identify patients with distant metastatic lung cancer was 58.3% and 88.2%, respectively, based on an analysis of Medicare claims data and information from the Surveillance, Epidemiology and End Results (SEER) program [14]. We note, however, that while Cooper and colleagues required only one Medicare claim with an ICD-9-CM code for diagnosis of secondary malignant neoplasm (i.e., metastatic disease), we required two such claims to increase the specificity of our case-ascertainment methods. We also note that the size and composition of the study population was largely robust when employing alternative sample-selection criteria (e.g., excluding all patients with evidence of multiple primary tumors, irrespective of their relationship with the site of metastasis [3% of study population]), and when considering other evidence for lung cancer (e.g., chemotherapy regimen) among patients with multiple documented primary tumor types. However, because the study algorithms have not been formally validated, their accuracy (and by implication, any resultant bias) is unknown. Finally, because we focused attention on the subgroup of patients with metastatic lung cancer who received chemotherapy, the results of our study may not be generalizable to all patients with metastatic lung cancer, including those who did not receive chemotherapy.

Several additional limitations of our study should be noted. First, our case-finding algorithm may have missed some patients receiving chemotherapy. It typically takes one year or more for newly approved products to receive their own HCPCS codes that can be used for billing purposes. During the intervening period, providers use nonspecific (or miscellaneous) codes that also may be used for other drugs. Second, given the terminal nature of metastatic lung cancer, health plan disenrollment in this study was assumed to occur as a result of death; disenrollment therefore was not treated as a "censoring event." Switching between health plans during treatment for metastatic disease should be an infrequent event. Third, as the perspective of our analysis was that of a third-party payer, we included neither out-of-pocket expenses and co-payments, nor indirect costs (i.e., the value of morbidity- and mortality-related productivity loss). Finally, we did not adjust payment amounts for inflation using a general or medical price index (since it may not be appropriate for this particular subset of patients -- in this particular subset of health plans -- who consumed specific healthcare services), and our estimates thus reflect the experience of patients in our sample from 2001 to 2006.

## Conclusion

The results of our study suggest that the economic burden of patients with metastatic lung cancer receiving chemotherapy is substantial -- exceeding $125,000. The results of our study also suggest that the majority of costs are associated with outpatient--rather than inpatient--care. Such findings may be important in informing the overall allocation of healthcare resources, in defining potential cost savings from disease prevention, and in evaluating the cost-effectiveness of new medical interventions.

## Competing interests

Gerry Oster, Montserrat Vera-Llonch, and Derek Weycker are employees of PAI, which received funding for this research from Amgen Inc. Andrew Glass is a practicing oncologist at The Center for Health Research, Kaiser Permanente Northwest, Portland, OR, and received funding for this research. Sue Gao and Beth Barber are employees of Amgen. Rohit Borker was an employee of Amgen at the time of the study; he is currently an employee of GlaxoSmithKline (One Franklin Plaza FP1280, 200 North 16^th ^Street, Philadelphia, PA 19102).

## Authors' contributions

All authors - MVL, DW, AG, SG, RB, BB, and GO - had significant contributions to study conception and design, development of study methodologies, analyses and discussion of study findings, manuscript development, and final editorial review.

## Pre-publication history

The pre-publication history for this paper can be accessed here:

http://www.biomedcentral.com/1472-6963/11/305/prepub

## Supplementary Material

Additional file 1 **Appendix**. Table. "Sample attrition: Metastatic lung cancer". Click here for file
