# Supplementary material for: Healthcare costs in patients with metastatic lung cancer receiving chemotherapy
Source: BMC Health Serv Res. 2011 Nov 10;11:305. doi: 10.1186/1472-6963-11-305 (PMC3239411; doi:10.1186/1472-6963-11-305)
Supplement: Additional file 1 — Appendix. Table. "Sample attrition: Metastatic lung cancer". [file 1472-6963-11-305-S1.PDF]

Appendix. Sample attrition: Metastatic lung cancer

Criteria		n	?
1)	Two or more lung cancer diagnoses	25,708	
2)	Two or more diagnoses of secondary malignant neoplasm	6,398	-19,310
3)	Two or more diagnoses of other primary malignant neoplasm 60 days or more before first evidence of secondary malignant neoplasm	6,289	-109
4)	- Include if site of metastasis is same as site of other primary	6,398	109
5)	Evidence of chemotherapy on or after first diagnosis of secondary malignant neoplasm or prior 45 days	6,057	-232
6)	Continuous enrollment 12 months prior to diagnosis of secondary malignant neoplasm	4,068	-1,989
